# Bilateral proptosis and bitemporal swelling: A rare manifestation of acute myeloid leukemia

**DOI:** 10.4103/1817-1745.66687

**Published:** 2010

**Authors:** Dinesh Rajput, Ram Naval, Kamlesh Yadav, Arun Tungaria, Sanjay Behari

**Affiliations:** Department of Neurosurgery, Sanjay Gandhi Postgraduate Institute of Medical Sciences, Lucknow - 226 014, India; 1 Department of Pathology, Sanjay Gandhi Postgraduate Institute of Medical Sciences, Lucknow - 226 014, India

**Keywords:** Acute myeloid leukemia, bitemporal swelling, chemotherapy, proptosis

## Abstract

**Background:**

In Acute Myeloid Leukemia (AML), malignant clones of immature myeloid cells (primarily blasts) proliferate, replace bone marrow, circulate in blood and invade other tissues. The unique presentation of bilateral proptosis and bilateral temporal swelling in AML is being reported.

**Case Report:**

A 6-year-old girl presented with low-grade fever, progressively increasing bitemporal swelling and bilateral proptosis. Contrast Enhanced Computed Tomographic (CECT) images revealed enhancing infiltrates occupying the lateral orbital wall, causing proptosis. The infiltrate extended toward the bilateral temporal fossae beneath the temporalis muscle and extradurally beneath the frontal and temporal bones. A high total leucocytic count with immature and deformed cells and, Fine Needle Aspiration Cytology (FNAC) from the temporal swelling, the bone marrow aspirate and biopsy showing leukemic blast cells confirmed the diagnosis of AML. Chemotherapy brought about remission of the disease.

**Conclusions:**

To the best of the authors’ knowledge, simultaneous presence of both bilateral proptosis and bitemporal swellings have not been previously reported in AML. A peripheral blood smear with bone marrow aspirate and biopsy help in the early detection of AML. Institution of early intervention in this potentially fatal disease is often associated with gratifying survival rates.

## Introduction

In Acute Myeloid Leukemia (AML), malignant clones of immature myeloid cells (primarily blasts) proliferate and eventually replace the bone marrow, circulate in blood and invade other tissues of the body. The usual manifestations are due to the suppression of normal hematopoiesis by leukemia. In this report, the unique presentation of bilateral proptosis resulting from orbital infiltration as well as bilateral temporal swelling by AML is being reported.

## Case Report

**Figure 1:**
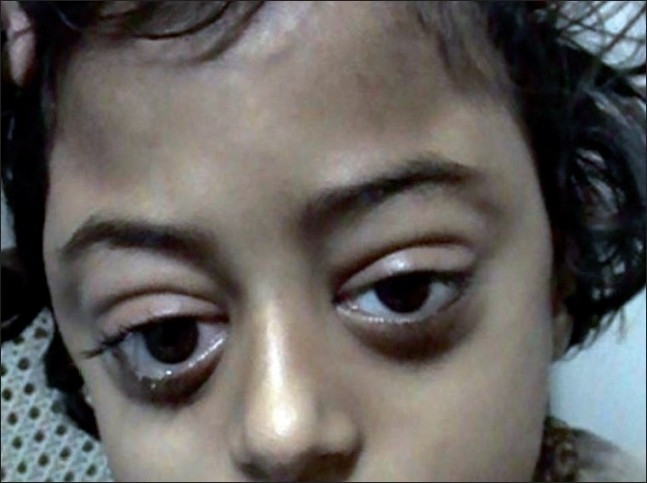
A 6-year-old female child showing bilateral proptosis and bitemporal swelling

**Figure 2:**
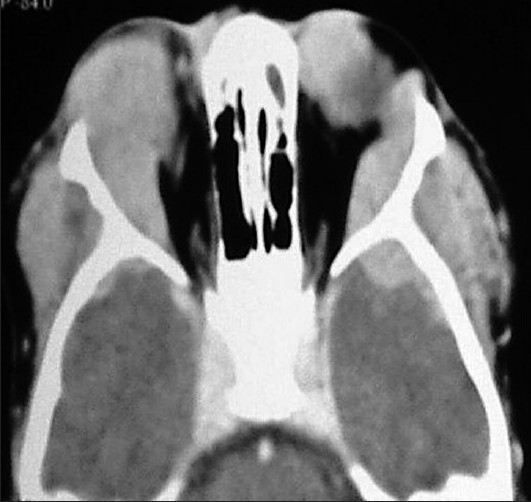
Axial CECT showing enhancing infiltrates occupying the lateral orbital wall and causing proptosis. The infiltrate extended toward the bilateral temporal fossae beneath the temporalis muscle. There were extradural infiltrates extending bilaterally extradurally beneath the temporal bones

**Figure 3:**
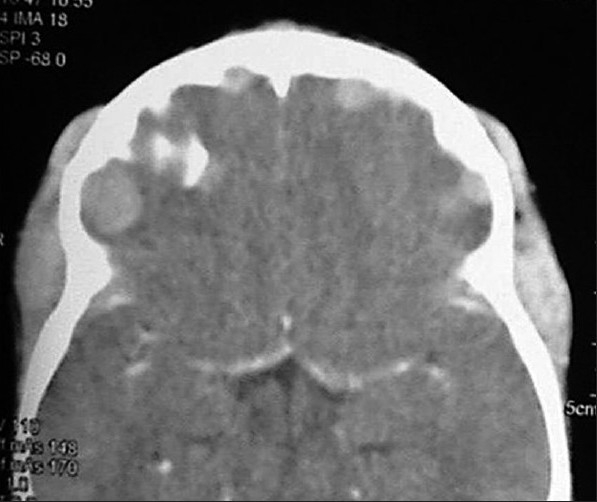
Axial CECT showing extradural infiltrates extending bilaterally beneath the frontal and temporal bones. On both sides, small lobules were extending into the cortex of the frontal lobes and causing perifocal edema

**Figure 4:**
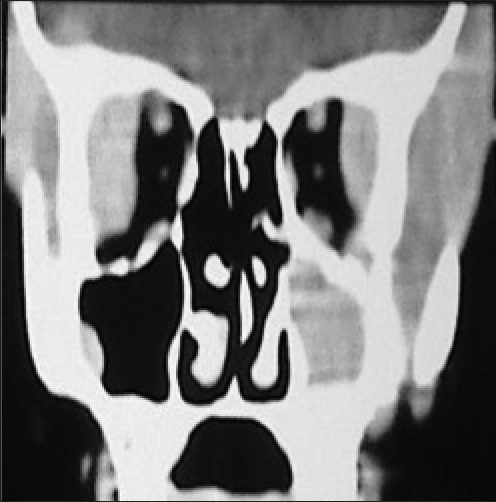
Coronal CT showed the left maxilla also infiltrated by the lesion

**Figure 5:**
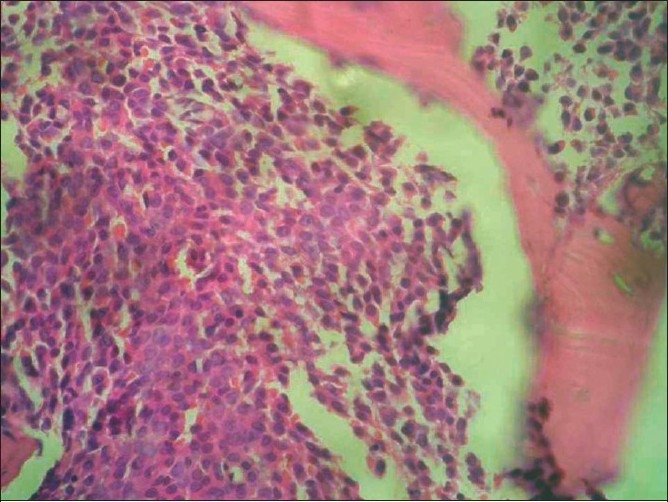
Bone marrow biopsy showing hypercellular marrow with sheets of blast cells (H & E, 40×)

**Figure 6:**
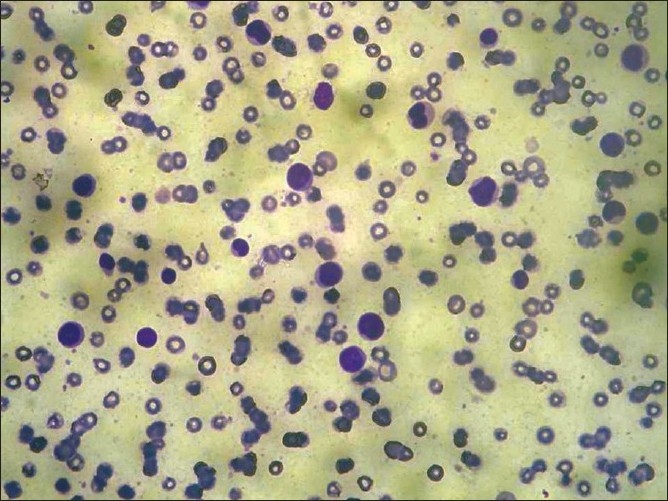
FNAC from temporal swelling with hemorrhagic background showing blast cells (May Grunwald Geimsa stain, 20×)

**Figure 7:**
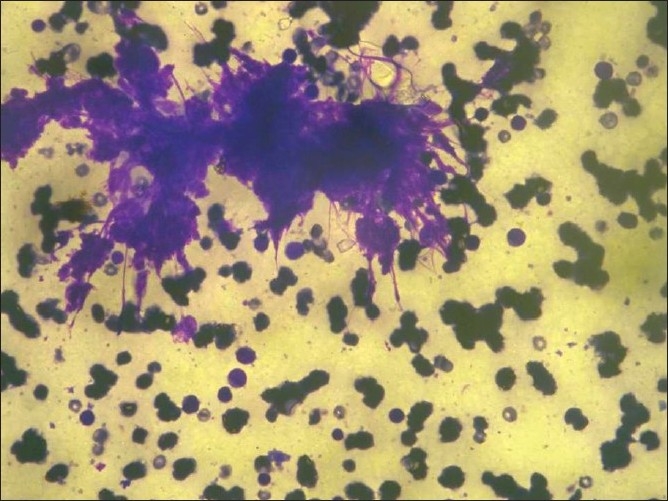
FNAC from temporal swelling showing clumped blast cells with an occasional signal blast cell (May Grunwald Geimsa stain, 20×)

**Figure 8:**
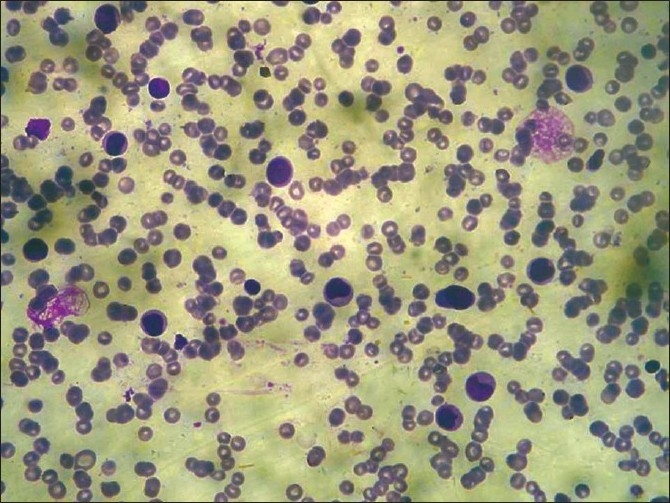
Peripheral blood smear showing blast cells (Giemsa stain, 40×)

A 6-year-old girl presented with low-grade fever for 1 month, with progressive increasing bitemporal swelling and bilateral proptosis (right greater than left) [Figure 1]. On examination, she was markedly cachexic and had significant loss of appetite and weight. She had bilateral proptosis with normal visual acuity and extraocular movements. There was an ill-defined, nontender, firm swelling in the bilateral temporal region with the skin over the swelling being normal. Her neurological examination was normal. There was no palpable abdominal organomegaly and no lymphadenopathy. The contrast-enhanced axial Computed Tomographic (CT) images showed an enhancing infiltrate occupying the lateral wall of the orbit pushing the globe outwards and manifesting as proptosis. The infiltrate extended toward the bilateral temporal fossae beneath the temporalis muscle. There were extradural infiltrates extending bilaterally beneath the frontal and temporal bones. On both sides, small lobules were extending into the cortex of the frontal lobes and causing perifocal edema [Figures 2 and 3]. The coronal CT showed that the left maxilla was also filled by the lesion [Figure 4]. Her hemoglobin was 9 gm% and the total leucocyte count was 34,800/mm^3^. Her differential leucocytic count showed 5% neutrophils, 14% lymphocytes and 1% reticulocytes. The rest of the cells were immature and deformed cells. The FNAC from the bitemporal swelling showed clusters of atypical cells with a high nuclear–cytoplasmic ratio. The cells contained an irregularly shaped nuclei, with two to three nucleoli and scanty cytoplasm. The cytology was suggestive of a leukemic infiltrate. The bone marrow aspirate and biopsy showed hypercellular marrow smears with proliferation of blasts (approximately 25–30%). The blasts were positive for myeloperoxidase; erythroid cells and megakaryocytes were reduced. The findings were suggestive of AML [Figures 5–8].

Following diagnosis, she undertook two cycles of intensive chemotherapy, including cytosine arabinoside (100 mg/m^2^) and doxorubicin (30 mg/m^2^) in a 7-day cycle at her local hospital, with consequent remission of the disease. She did not report for a subsequent follow-up at our center.

## Discussion

AML accounts for approximately 15% of all leukemias in children.[1] Leukemic cells may infiltrate any extramedullary site. Their accumulation in soft tissue or bone is termed as granulocytic sarcoma, an uncommon presentation, occurring in approximately 3% of patients with AML.[2] This is also termed as chloroma,[3] because leukemic cells containing myeloperoxidase turn green when exposed to ultraviolet light. AML most commonly affects children and young adults, the median age at presentation being 7 years.[4]

Leukemic cells originate in the bone marrow.[1] In the head and neck, they involve the skull, orbits and paranasal sinuses.[1] Our patient had the unique presentation of bilateral proptosis with bitemporal swelling. The tumor had also infiltrated the frontotemporal extra- and intradural compartments and the paranasal sinuses. The presence of unilateral and bilateral proptosis has been reported with AML.[4–6] The proptosis in these cases is mainly due to leukemic infiltrates, retrobulbar hemorrhage, orbital muscle infiltration or venous blockage. In a study, AML was associated with 9.3% orbital masses.[5] Other presentations due to orbital involvement include ptosis, lacrimal gland involvement, conjuctival masses, iridic and diffuse uveal involvement.[2,6] Most of the reported cases have decreased visual acuity and restricted extra-ocular movements. Our patient was different in having normal visual acuity and eyeball movements perhaps due to an early detection of the leukemia as well as extension of the leukemic infiltration toward the bilateral temporal fossae rather than within the orbit.

She also had bitemporal swelling that was firm, not tender and had molded into the inner and outer surfaces of bilateral orbital walls without causing any destruction. Although unilateral temporal swelling with AML has also been described in the literature,[7] to the best of our knowledge, the simultaneous presence of both bilateral proptosis and bitemporal swellings have not been previously reported in AML. Other infiltrative lesions like non-Hodgkin lymphoma[8] or eosinophilic granuloma[9] or other temporal bone tumors, developmental lesions like arachnoid cyst,[10] fibrous dysplasia[11] and neurofibromatosis with sphenoid wing dysplasia[12] are often unilateral and usually not associated with bilateral proptosis.

Peripheral smear is an invaluable tool in diagnosing the systemic form of AML showing immature blast cells with a high total leukocyte count and relative neutropenia. Leukemic proptosis, however, may not always be associated with leukocytosis or immature cells in the peripheral smear. Doing a peripheral smear along with bone marrow aspirate and biopsy in all patients of AML manifesting with proptosis in the pediatric age is, therefore, justified. Immunohistochemistry and immunocytochemistry are used to detect the antibody against myeloperoxidase. On CT scan, the extramedullary leukemia within the orbits may appear as a well defined lesion, isodense to muscles[1] and enhancing on contrast, usually confined to the orbits but occasionally also extending to the extradural temporal fossae, temporal and frontal regions and rarely also to the paranasal sinuses, as seen in our patient. On magnetic resonance imaging, the lesion is isointense on T1-weighted and hyperintense on T2-weighted image, with intense enhancement on contrast.[1,3,6] The prognosis depends on the course of underlying systemic malignancy. The presence of extramedullary leukemia does not alter the survival of patients with AML.[6]

Chemotherapy is the mainstay of treatment.[1,6] Chemotherapy includes both an intensive and consolidation phase. In the intensive phase, the drugs doxorubicin and cytosine arabinocide are used. Bone marrow suppression is strictly monitored with the patients being kept in isolation and being administered proper supportive treatment. Growth factor helps in stimulating marrow following the intensive phase of chemotherapy. At the end of 4 weeks, bone marrow aspirate and biopsy should be repeated to assess for remission. If remission has not been achieved, the same cycle of chemotherapy is repeated. In case remission has been achieved, the consolidation phase starts in the form of cytosine arabinoside (over a period of 4–8 months).[13] Despite the advances in chemotherapeutic schedules, an allogenic bone marrow transplantation from a matched family donor still remains the best long-term option that provides remission-free survival for most patients.[13]

## Conclusion

AML should be kept in the differential diagnosis of a child presenting with proptosis or orbital mass with or without skull lesions. A peripheral blood smear should be performed in all cases along with bone marrow aspirate and biopsy for an early detection of AML. Institution of early intervention in this potentially fatal disease is often associated with gratifying 5-year survival rates.
